# A Prospective Study of Quality of Life After an Ambulatory Visit for Heart Failure

**DOI:** 10.1016/j.yjcafi.2025.10.003

**Published:** 2026-01-21

**Authors:** ANDREW P. AMBROSY, RISHI V. PARIKH, ELISHA A. GARCIA, KELLY M. WETZEL, ALAN S. GO

**Affiliations:** 1 Department of Cardiology, Kaiser Permanente San Francisco Medical Center, San Francisco, CA;; 2 Division of Research, Kaiser Permanente Northern California, Pleasanton, CA;; 3 Department of Health Systems Science, Kaiser Permanente Bernard J. Tyson School of Medicine, Pasadena, CA; 4 Departments of Epidemiology, Biostatistics and Medicine, University of California, San Francisco, San Francisco, CA.

## Introduction

Overall survival rates for ambulatory patients with heart failure (HF) have improved with the uptake of guideline-directed medical therapy, but impairments in quality of life (QoL) and functional capacity persist.^1^ Patients hospitalized due to HF often experience severe and prolonged declines in QoL, with symptom burdens comparable to those seen in debilitating conditions, such as acute myocardial infarction, end-stage kidney disease, and common malignancies.^2,3^ Furthermore, prior studies suggest that patients with HF often value improvements in QoL over extended survival.^4^ Despite these insights, the trajectory of QoL in the ambulatory HF population, particularly following emergency department (ED) or clinic visits, remains poorly described. To address this important knowledge gap, we conducted a large, cross-sectional, survey-based study to evaluate both disease-specific and generic QoL among patients recently seen for HF in the ED or clinic within a demographically diverse cohort.

## Methods

We conducted a cross-sectional observational survey study of patients with recent ED or outpatient clinic visits due to HF. Eligible participants were identified through their electronic health records (EHRs), based on having a qualifying encounter for HF within the previous 3 months. Exclusion criteria were prior heart transplantation or presence of a left ventricular assist device, end-stage kidney disease requiring chronic dialysis or kidney transplant, or an anticipated life expectancy of less than 1 year. The study was approved by the Kaiser Permanente Northern California Institutional Review Board.

Participants were invited to complete the survey via secure e-mail, with up to 2 reminder e-mails sent at 24 and 72 hours after the initial invitation. Surveys were administered by using a secure and validated instance of REDCap. QoL was assessed by using 2 validated instruments. The Kansas City Cardiomyopathy Questionnaire (KCCQ-12) is a 12-item, disease-specific instrument that evaluates HF symptoms, physical function, social limitations, and QoL, with scores ranging from 0 100, where higher scores indicate better status.^5^ The EuroQol-5 Dimensions (EQ-5D) is a generic health-status measurement that includes 5 domains (mobility, self-care, usual activities, pain/discomfort, and anxiety/depression), along with a visual analogue scale (VAS), scored from 0 100.^6^ The EQ-5D was summarized into a single utility score using the U.S. value set, which weights the responses to each domain based on the health-state preferences of the general population.^7^

Descriptive statistics were used to summarize the baseline characteristics and QoL scores. Group differences between patients seen in the ED vs the outpatient clinic were assessed using *t* tests or Wilcoxon rank-sum tests for continuous variables and^2^ or Fisher exact tests for categorical variables. Crude rates per 100 person-years and associated 95% Poisson intervals were calculated for each outcome. Poisson regression was used to calculate the rate ratio and 95% CI for each QoL score (per standard deviation) and outcome. Differences in the rate ratio for QoL scores between patients seen in the ED vs the outpatient clinic were assessed by using an interaction term. All analyses were performed using SAS, version 9.4.

## Results

Of the 6983 individuals contacted, 1056 completed the survey, yielding a response rate of 15.1%. The final analytical cohort included the first 1000 responses (Table 1). Among these, 211 patients had had a recent ED visit due to HF, and 789 patients had had a recent outpatient clinic visit. The mean age was 75 ± 11 years; 41% were female, and 79% identified as non-Hispanic white. The median (25th, 75th) left ventricular ejection fraction (LVEF) was 55 (45, 60), reflecting a predominance of patients with mildly reduced or preserved LVEF.

**Fig. 1.**
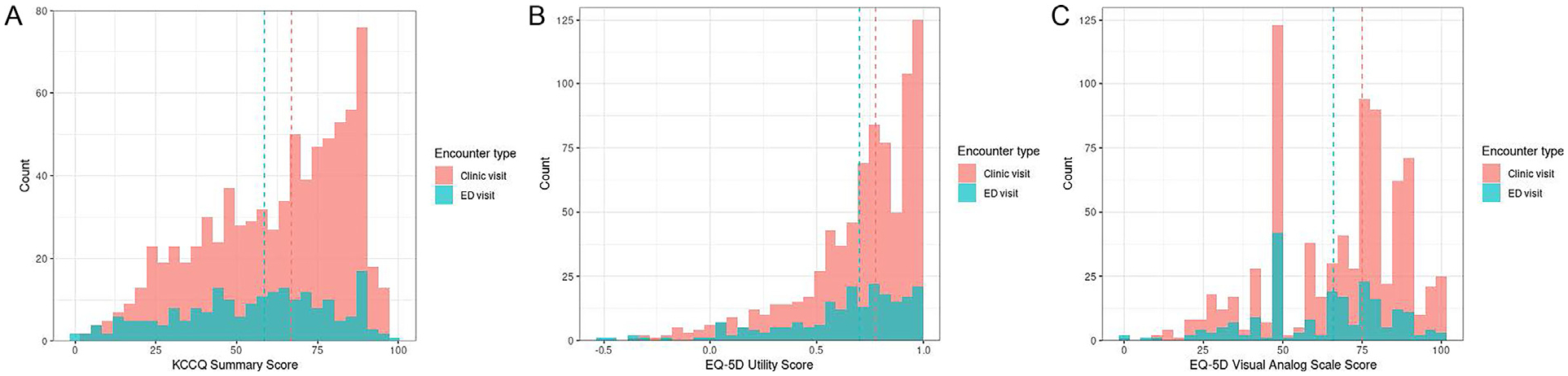
Histogram showing A, the distribution of Kansas City Cardiomyopathy Questionnaire overall summary scores; B, EuroQol-5 Dimension descriptive index scores; and C, EuroQol-5 Dimension Visual Analogue Scale scores.

The median (IQR) KCCQ score in the cohort was 64 (44,80). Notably, patients seen in the ED had significantly lower KCCQ scores compared to those seen in the outpatient clinic, with median scores of 59 (38, 73) vs 67 (46,82), respectively (*P* < 0.001) (Fig. 1). Similarly, the EQ-5D utility score was lower in patients seen in the ED, with a median score of 0.7 (0.5, 0.8), compared to 0.8 (0.6, 0.9) in those seen in the clinic (*P* < 0.001). The EQ-5D VAS scores followed a similar pattern, with a cohort-wide median of 71 (50, 81) and significantly lower scores in the ED patients (66, IQR 50, 78) compared to the clinic patients (75, IQR 50, 83) (*P* < 0.001). In subgroup analyses by LVEF phenotype, patients with HFpEF (LVEF ≥ 50%; KCCQ 63 [IQR 42–79], EQ-5D utility 0.7 [0.6 0.9], EQ-5D VAS 70 [50–80]) reported marginally lower KCCQ and EQ-5D utility scores than those with HFrEF (≤ 40%; 66 [45–81], 0.8 [0.6–0.9], 74 [50 85]) or HFmrEF (41 49%; 67 [47–83], 0.8 [0.6–0.9], 75 [50–85]), but similar EQ-5D VAS scores.

Compared with ED visits, clinic visits were associated with lower unadjusted rates (per 100 person-years) of hospitalization for HF as primary diagnoses (4.6 vs 10.6), all-cause hospitalization (35.1 vs 78.1), and all-cause death (7.1 vs 7.7). Lower QoL scores were significantly associated with an increased risk of adverse outcomes over the 6-month follow-up period. Patients with lower KCCQ and EQ-5D utility scores experienced higher rates of all outcomes (Table 2). These associations persisted after adjustment for age, sex, comorbidities, and other relevant covariates. Of note, the KCCQ and EQ-5D scores were strong and consistent predictors of adverse outcomes. Point estimates for QoL scores across all outcomes were stronger among patients seen in outpatient clinics compared to the ED, though these differences were not statistically significant (Table 2).

## Discussion

To our knowledge, this study represents the largest contemporary survey of disease-specific and generic QoL among patients with HF after outpatient or ED clinical encounters. Despite being largely managed in ambulatory settings and treated with evidence-based HF therapies, many patients reported persistently impaired QoL. Importantly, patients with HFpEF reported marginally lower QoLs compared with those with reduced or mildly reduced EF, consistent with the more advanced ages and multimorbidities of this subgroup. Based on KCCQ scores, the majority of participants fell into the categories of poor-to-fair or fair-to-good health status, indicating substantial room for improvement in their day-to-day experiences of living with HF.

It is notable that patients seen in the ED reported significantly worse QoL across all survey instruments compared to those seen in the clinic. This difference may reflect a more acutely symptomatic population or individuals with poorer access to longitudinal care, and it underscores the importance of understanding clinical context when interpreting patient-reported outcomes. Additionally, our findings demonstrate that worse QoL scores are strongly and consistently associated with long-term outcomes, including hospitalization and death, regardless of clinical setting. These observations reinforce the prognostic usefulness of QoL assessment, and they support its role as a valuable component of routine outpatient HF care.^8^ In particular, stratifying patients by QoL score could help to identify those at highest risk for adverse outcomes, enabling clinicians to prioritize follow-up intensity, tailor supportive services, and intervene earlier in the disease trajectory.

In the ambulatory setting, QoL appears to be dynamic and may fluctuate in response to symptoms, decompensation, or therapeutic intervention. As such, serial measurement of QoL could provide meaningful insight into disease trajectory and aid in risk stratification or treatment planning. However, despite its prognostic value, few HF therapies meaningfully improve QoL. While IV iron therapy and cardiac resynchronization therapy in selected patients have demonstrated benefits in this regard, most pharmacological treatments exert greater effects on clinical rather than on patient-reported outcomes.^1^ These results highlight the ongoing need for treatments that specifically target symptoms and functional status.

### Limitations

This study has several limitations. First, survey-based research is subject to response and selection bias, particularly given the reliance on electronic communication for recruitment. As such, digitally underserved patients—including older adults and those with lower educational attainment—may have been underrepresented in our sample, which could limit generalizability. Second, because this was a cross-sectional assessment, we could not evaluate changes in QoL over time. Third, although we adjusted for multiple clinical and demographic covariates, unmeasured confounding remains possible. Additionally, because our study did not include a comparator group of older adults without HF, we could not directly quantify the degree to which QoL impairment exceeded that expected based on age alone.

## Conclusions

In conclusion, we found that QoL among patients with HF remains impaired in the outpatient setting, particularly following ED visits. Worse QoL scores were independently associated with increased risks of hospitalization and death. These findings suggest a potential role for routine QoL assessment in identifying high-risk patients and informing clinical care. As HF management continues to evolve, attention to the lived experience of patients—including symptoms, function, and QoL—must remain central to improving patient-centered outcomes.

## Figures and Tables

**Table 1:** Baseline clinical characteristics of the overall cohort, stratified by type of clinical encounter (emergency department vs outpatient clinic visit)

Characteristics	Overall (N=1000)	ED visit (N=211)	Ambulatory visit (N=789)	*P* Value
Demographics				
Age, years	75 (11)	74 (13)	75 (10)	0.48
Female, n (%)	411 (41.1)	95 (45.0)	316 (40.1)	0.19
Self-reported race, n (%)				0.11
American Indian/Alaska Native	1 (0.1)	0 (0.0)	1 (0.1)	
Asian	72 (7.2)	14 (6.6)	58 (7.4)	
Black	52 (5.2)	13 (6.2)	39 (4.9)	
Hawaiian/Pacific Islander	2 (0.2)	2 (0.9)	0 (0.0)	
Multiracial	37 (3.7)	11 (5.2)	26 (3.3)	
White	792 (79.2)	161 (76.3)	631 (80.0)	
Unknown	44 (4.4)	10 (4.7)	34 (4.3)	
Hispanic ethnicity, n (%)	68 (6.8)	19 (9.0)	49 (6.2)	0.15
Medical History				
Atrial fibrillation or flutter	570 (57.0)	128 (60.7)	442 (56.0)	0.23
Ventricular fibrillation or tachycardia	55 (5.5)	18 (8.5)	37 (4.7)	<0.05
Ischemic stroke or transient ischemic attack	70 (7.0)	19 (9.0)	51 (6.5)	0.2
Acute myocardial infarction	87 (8.7)	20 (9.5)	67 (8.5)	0.65
Mitral or aortic valvular disease	328 (32.8)	72 (34.1)	256 (32.4)	0.64
Peripheral artery disease	101 (10.1)	24 (11.4)	77 (9.8)	0.49
Diabetes mellitus	644 (64.4)	139 (65.9)	505 (64.0)	0.61
Hypertension	817 (81.7)	175 (82.9)	642 (81.4)	0.6
Dyslipidemia	864 (86.4)	178 (84.4)	686 (86.9)	0.33
Chronic kidney disease	517 (51.7)	119 (56.4)	398 (50.4)	0.12
Chronic liver disease	67 (6.7)	11 (5.2)	56 (7.1)	0.33
Chronic lung disease	482 (48.2)	110 (52.1)	372 (47.1)	0.2
Cardiac Intervention History				
Coronary artery bypass graft	58 (5.8)	9 (4.3)	49 (6.2)	0.28
Percutaneous coronary intervention	107 (10.7)	27 (12.8)	80 (10.1)	0.27
Implantable cardioverter defibrillator	85 (8.5)	17 (8.1)	68 (8.6)	0.79
Cardiac resynchronization therapy	1 (0.1)	0 (0.0)	1 (0.1)	0.6
Vital Signs				
Body mass index, kg/m^2^	30 (7)	30 (9)	30 (7)	0.37
Systolic blood pressure, mmHg	127 (22)	127 (24)	126 (21)	0.62
Heart rate, bpm	74 (15)	78 (18)	73 (14)	<0.001
Medications				
Angiotensin-converting enzyme inhibitor	203 (20.3)	42 (19.9)	161 (20.4)	0.87
Angiotensin II receptor blocker	328 (32.8)	73 (34.6)	255 (32.3)	0.53
Angiotensin-neprilysin inhibitor	225 (22.5)	34 (16.1)	191 (24.2)	<0.05
Mineralocorticoid receptor antagonist	418 (41.8)	90 (42.7)	328 (41.6)	0.78
Diuretic	649 (64.9)	151 (71.6)	498 (63.1)	<0.05
Beta-blocker	800 (80.0)	164 (77.7)	636 (80.6)	0.35
SGLT2 inhibitor	446 (44.6)	86 (40.8)	360 (45.6)	0.21
Vasodilator	198 (19.8)	51 (24.2)	147 (18.6)	0.07
Nitrate	141 (14.1)	36 (17.1)	105 (13.3)	0.16
Laboratory Values				
Left ventricular ejection fraction, %	50 (12)	51 (14)	51 (12)	0.96
Median (q1, q3)	55 (45, 60)	55 (40, 60)	55 (45, 60)	0.77
Hemoglobin, g/dL	13.5 (1.9)	13.1 (2.1)	13.5 (1.8)	<0.05
Hemoglobin A1C, %	6.2 (1.1)	6.4 (1.3)	6.2 (1.0)	<0.05
Serum creatinine, mg/dL	1.2 (0.5)	1.3 (0.6)	1.2 (0.5)	0.07
Estimated glomerular filtration rate, mL/min/1.73m2	62 (21)	60 (22)	63 (20)	0.07
Converted Urine Albumin-to-Creatinine Ratio Category				0.35
< 30	245 (24.5)	43 (20.4)	202 (25.6)	
30–299	61 (6.1)	16 (7.6)	45 (5.7)	
300+	42 (4.2)	8 (3.8)	34 (4.3)	
Unknown	652 (65.2)	144 (68.2)	508 (64.4)	
KCCQ Responses				
Summary core (0–100)	60.5 (22.9)	54.7 (24.1)	62.1 (22.3)	<0.001
Median (q1, q3)	64.4 (43.8, 80.1)	58.5 (37.7,72.9)	66.9 (45.6, 81.7)	<0.001
Physical Limitation Score (0–100)	53.6 (23.3)	49.6 (24.2)	54.7 (23.0)	<0.01
Median (q1, q3)	53.3 (33.3, 73.3)	53.3 (33.3, 66.7)	53.3 (33.3, 73.3)	<0.01
Symptom frequency score (0–100)	70.0 (25.1)	65.1 (25.5)	71.4 (24.8)	<0.01
Median (q1, q3)	75.0 (50.0, 90.0)	65.0 (45.0, 85.0)	80.0 (55.0, 95.0)	<0.001
Quality-of-life score (0–100)	63.2 (29.4)	55.2 (30.5)	65.4 (28.7)	<0.001
Median (q1, q3)	62.5 (37.5, 87.5)	50.0 (37.5, 87.5)	75.0 (50.0, 87.5)	<0.001
Social limitation score (0–100)	55.1 (26.0)	48.8 (27.7)	56.8 (25.2)	<0.001
Median (q1, q3)	60.0 (33.3, 80.0)	53.3 (20.0, 73.3)	60.0 (40.0, 80.0)	<0.001
Physical limitations-showering/bathing	4.4 (1.0)	4.2 (1.2)	4.5 (1.0)	<0.01
Median (q1, q3)	5.0 (4.0, 5.0)	5.0 (4.0, 5.0)	5.0 (4.0, 5.0)	<0.01
Physical limitation-walking 1 block	3.8 (1.4)	3.6 (1.5)	3.9 (1.4)	<0.01
Median (q1, q3)	4.0 (3.0, 5.0)	4.0 (2.0, 5.0)	4.0 (3.0, 5.0)	<0.01
Physical limitation-hurrying/jogging	2.8 (1.8)	2.6 (1.8)	2.9 (1.8)	0.13
Median (q1, q3)	2.0 (1.0, 4.0)	2.0 (1.0, 4.0)	2.0 (1.0, 4.0)	0.13
Symptom frequency-swelling	4.0 (1.4)	3.8 (1.4)	4.0 (1.4)	0.11
Median (q1, q3)	5.0 (3.0, 5.0)	4.0 (3.0, 5.0)	5.0 (3.0, 5.0)	<0.05
Symptom frequency-fatigue	4.4 (2.0)	4.1 (2.0)	4.5 (2.0)	<0.01
Median (q1, q3)	5.0 (3.0, 6.0)	4.0 (2.0, 6.0)	5.0 (3.0, 6.0)	<0.01
Symptom frequency-SOB	5.1 (2.0)	4.6 (2.1)	5.2 (2.0)	<0.001
Median (q1, q3)	6.0 (3.0, 7.0)	5.0 (3.0, 7.0)	6.0 (3.0, 7.0)	<0.001
Symptom frequency-sleep sitting up	4.5 (1.1)	4.4 (1.1)	4.6 (1.1)	0.15
Median (q1, q3)	5.0 (5.0, 5.0)	5.0 (4.0, 5.0)	5.0 (5.0, 5.0)	<0.05
Quality of life-enjoyment	3.8 (1.2)	3.5 (1.3)	3.9 (1.2)	<0.001
Median (q1, q3)	4.0 (3.0, 5.0)	4.0 (3.0, 5.0)	4.0 (3.0, 5.0)	<0.001
Quality of life-satisfaction	3.3 (1.3)	2.9 (1.3)	3.4 (1.3)	<0.001
Median (q1, q3)	3.0 (2.0, 4.0)	3.0 (2.0, 4.0)	4.0 (2.0, 4.0)	<0.001
Social limitation- hobbies/recreation	3.6 (1.5)	3.3 (1.5)	3.7 (1.5)	<0.001
Median (q1, q3)	4.0 (2.0, 5.0)	3.0 (2.0, 4.0)	4.0 (2.0, 5.0)	<0.001
Social limitation-work/chores	3.6 (1.4)	3.3 (1.5)	3.7 (1.4)	<0.001
Median (q1, q3)	4.0 (2.0, 5.0)	3.0 (2.0, 4.0)	4.0 (3.0, 5.0)	<0.001
Social limitation-visit family/friends	4.1 (1.4)	3.8 (1.5)	4.1 (1.3)	<0.01
Median (q1, q3)	5.0 (3.0, 5.0)	4.0 (3.0, 5.0)	5.0 (3.0, 5.0)	<0.01
EuroQol-5D Responses				
EQ5D Utility Score (USA Value Set)	0.7 (0.3)	0.6 (0.3)	0.7 (0.3)	<0.001
Median (q1, q3)	0.8 (0.6, 0.9)	0.7 (0.5, 0.8)	0.8 (0.6, 0.9)	<0.001
Level summary score	9.4 (3.8)	10.3 (4.1)	9.2 (3.7)	<0.001
Median (q1, q3)	9.0 (6.0,12.0)	10.0 (7.0,13.0)	8.0 (6.0,11.0)	<0.001
Mobility	2.2 (1.1)	2.3 (1.1)	2.1 (1.1)	<0.01
Median (q1, q3)	2.0 (1.0, 3.0)	2.0 (1.0, 3.0)	2.0 (1.0, 3.0)	<0.01
Self-care	1.4 (0.8)	1.5 (0.9)	1.4 (0.8)	<0.05
Median (q1, q3)	1.0 (1.0, 2.0)	1.0 (1.0, 2.0)	1.0 (1.0,1.0)	<0.05
Usual activities	2.2 (1.0)	2.4 (1.1)	2.1 (1.0)	<0.001
Median (q1, q3)	2.0 (1.0, 3.0)	2.0 (2.0, 3.0)	2.0 (1.0, 3.0)	<0.001
Pain/discomfort	1.9 (1.0)	2.1 (1.1)	1.9 (1.0)	<0.01
Median (q1, q3)	2.0 (1.0, 3.0)	2.0 (1.0, 3.0)	2.0 (1.0, 2.0)	<0.01
Anxiety/depression	1.8 (1.0)	2.0 (1.1)	1.7 (0.9)	<0.01
Median (q1, q3)	2.0 (1.0, 2.0)	2.0 (1.0, 3.0)	1.0 (1.0, 2.0)	<0.01
Visual analog scale score	66.6 (20.4)	62.6 (20.4)	67.7 (20.2)	<0.01
Median (q1, q3)	71.0 (50.0, 81.0)	66.0 (50.0, 78.0)	75.0 (50.0, 83.0)	<0.001

bpm, beats per minute; ED, emergency department; EQ-5D, EuroQol-5 Dimensions;. KCCQ, Kansas City Cardiomyopathy Questionnaire;;N, number; SGLT2, sodium-glucose cotranporter-2.

**Table 2:** Association between quality-of-life scores and subsequent heart failure hospitalizations, all-cause hospitalizations, and all-cause mortality

	KCCQ Summary Score per SD decrease (22.91 points)			
Outcome	No Interaction Model Overall RR	Interaction Model		
		ED RR	AVRR	Interaction
Unadjusted				
HF hospitalizations (primary dx)	1.39 (0.98,1.98)	1.02 (0.58,1.79)	1.58 (1.01,2.48)	0.23
HF hospitalizations (any dx)	1.45 (1.28,1.65)	0.95 (0.52,1.72)	1.47 (0.92, 2.33)	0.25
All-cause hospitalizations	1.41 (1.24,1.59)	0.99 (0.53,1.83)	1.49 (0.93, 2.40)	0.28
All-cause deaths	2.93 (1.80,4.77)	1.15 (0.93,1.42)	1.56 (1.33,1.84)	0.02
Composite of primary HF hospitalization and death	1.82 (1.39,2.40)	1.14 (0.92,1.41)	1.55 (1.31,1.83)	0.03
Adjusted for Age and Sex				
HF hospitalizations (primary dx)	1.29 (0.90,1.87)	1.16 (0.93,1.45)	1.53 (1.29,1.82)	0.05
HF hospitalizations (any dx)	1.44 (1.27,1.64)	1.17 (0.95,1.44)	1.47 (1.26,1.72)	0.08
All-cause hospitalizations	1.40 (1.23,1.58)	1.17 (0.95,1.44)	1.46 (1.24,1.71)	0.10
All-cause death	2.72 (1.64,4.53)	1.18 (0.95,1.46)	1.44 (1.22,1.69)	0.15
Composite of primary HF hospitalization and death	1.70 (1.27,2.26)	2.20 (1.11,4.35)	3.31 (1.66, 6.62)	0.41
Adjusted for cCinical Covariates*				
HF hospitalizations (primary dx)	1.32 (0.91,1.94)	2.11 (1.04,4.30)	3.11 (1.53, 6.33)	0.45
HF hospitalizations (any dx)	1.43 (1.25,1.64)	2.23 (1.04,4.76)	3.31 (1.61,6.79)	0.46
All-cause hospitalizations	1.39 (1.21,1.58)	1.40 (0.92, 2.13)	2.02 (1.40, 2.91)	0.20
All-cause deaths	2.90 (1.72,4.90)	1.33 (0.85, 2.07)	1.87 (1.28, 2.73)	0.24
Composite of primary HF hospitalizations and deaths	1.77 (1.31,2.39)	1.39 (0.88, 2.20)	1.95 (1.32, 2.87)	0.26
EuroQol-5D Utility Score per SD Decrease (0.28)				
Unadjusted				
HF hospitalizations (primary dx)	1.28 (0.94,1.75)	0.95 (0.54,1.66)	1.45 (0.99, 2.13)	0.21
HF hospitalizations (any dx)	1.35 (1.21,1.51)	1.20 (1.00,1.43)	1.37 (1.19,1.59)	0.25
All-cause hospitalizations	1.32 (1.19,1.48)	1.19 (1.00,1.42)	1.34 (1.17,1.54)	0.30
All-cause deaths	1.94 (1.41,2.68)	1.57 (0.98, 2.52)	2.16 (1.38,3.36)	0.33
Composite of primary HF hospitalization and death	1.54 (1.24,1.91)	1.22 (0.86,1.74)	1.70 (1.27, 2.26)	0.15
Adjusted for Age and Sex				
HF hospitalizations (primary dx)	1.26 (0.89,1.77)	0.93 (0.49,1.74)	1.41 (0.94, 2.14)	0.25
HF hospitalizations (any dx)	1.35 (1.20,1.51)	1.21 (1.01,1.45)	1.37 (1.18,1.58)	0.31
All-cause hospitalizations	1.32 (1.19,1.48)	1.21 (1.01,1.44)	1.34 (1.16,1.54)	0.38
All-cause deaths	2.11 (1.48,3.02)	1.74 (1.04, 2.94)	2.35 (1.43,3.87)	0.41
Composite of primary HF hospitalizations and deaths	1.56 (1.23,2.00)	1.27 (0.85,1.90)	1.71 (1.25, 2.34)	0.25
Adjusted for Clinical Covariates*				
HF hospitalizations (primary dx)	1.31 (0.92,1.84)	1.03 (0.55,1.94)	1.43 (0.95, 2.15)	0.38
HF hospitalizations (any dx)	1.31 (1.16,1.48)	1.19 (0.98,1.44)	1.34 (1.15,1.56)	0.33
All-cause hospitalizations	1.29 (1.15,1.45)	1.17 (0.98,1.42)	1.31 (1.13,1.52)	0.37
All-cause deaths	2.13 (1.49,3.05)	1.82 (1.04,3.21)	2.31 (1.43,3.74)	0.53
Composite of primary HF hospitalization and death	1.61 (1.23,2.06)	1.39 (0.92, 2.09)	1.72 (1.27, 2.33)	0.40

* Atherosclerotic cardiovascular disease, diabetes, chronic kidney disease, body mass index, systolic blood pressure, left ventricular ejection fraction, tobacco use.dx, diagnosis; ED, emergency department; HF, heart failure.
